# *CLCN1* Mutations in Czech Patients with Myotonia Congenita, *In Silico* Analysis of Novel and Known Mutations in the Human Dimeric Skeletal Muscle Chloride Channel

**DOI:** 10.1371/journal.pone.0082549

**Published:** 2013-12-11

**Authors:** Daniela Skálová, Jana Zídková, Stanislav Voháňka, Radim Mazanec, Zuzana Mušová, Petr Vondráček, Lenka Mrázová, Josef Kraus, Kamila Réblová, Lenka Fajkusová

**Affiliations:** 1 Centre of Molecular Biology and Gene Therapy, University Hospital, Brno, Brno, Czech Republic; 2 Central European Institute of Technology, Masaryk University, Brno, Czech Republic; 3 Department of Neurology, University Hospital Brno, Brno, Czech Republic; 4 Department of Neurology, Charles University Second Faculty of Medicine and University Hospital Motol, Prague, Czech Republic; 5 Department of Biology and Medical Genetics, Charles University Second Faculty of Medicine and University Hospital Motol, Prague, Czech Republic; 6 Department of Child Neurology, University Hospital Brno, Brno, Czech Republic; 7 Department of Child Neurology, Second School of Medicine, Charles University and University Hospital Motol, Prague, Czech Republic; Cinvestav-IPN, Mexico

## Abstract

Myotonia congenita (MC) is a genetic disease caused by mutations in the skeletal muscle chloride channel gene (*CLCN1*) encoding the skeletal muscle chloride channel (ClC-1). Mutations of *CLCN1* result in either autosomal dominant MC (Thomsen disease) or autosomal recessive MC (Becker disease). The ClC-1 protein is a homodimer with a separate ion pore within each monomer. Mutations causing recessive myotonia most likely affect properties of only the mutant monomer in the heterodimer, leaving the wild type monomer unaffected, while mutations causing dominant myotonia affect properties of both subunits in the heterodimer. Our study addresses two points: 1) molecular genetic diagnostics of MC by analysis of the *CLCN1* gene and 2) structural analysis of mutations in the homology model of the human dimeric ClC-1 protein. In the first part, 34 different types of *CLCN1* mutations were identified in 51 MC probands (14 mutations were new). In the second part, on the basis of the homology model we identified the amino acids which forming the dimer interface and those which form the Cl^-^ ion pathway. In the literature, we searched for mutations of these amino acids for which functional analyses were performed to assess the correlation between localisation of a mutation and occurrence of a dominant-negative effect (corresponding to dominant MC). This revealed that both types of mutations, with and without a dominant-negative effect, are localised at the dimer interface while solely mutations without a dominant-negative effect occur inside the chloride channel. This work is complemented by structural analysis of the homology model which provides elucidation of the effects of mutations, including a description of impacts of newly detected missense mutations.

## Introduction

Myotonia congenita (MC) is a skeletal muscle disorder that causes myotonia, an abnormal delay in muscle relaxation after voluntary or evoked muscle contraction, as the prominent symptom. MC is due to mutations in the skeletal muscle chloride channel gene (*CLCN1*) that is located on chromosome 7q35, encompasses 35 kb of genomic DNA, contains 23 exons, and encodes the skeletal muscle chloride channel ClC-1 [1,2]. Mutations of *CLCN1* result in either autosomal dominant (Thomsen disease) or autosomal recessive (Becker disease) MC, and a subset have been found to cause both recessive and dominant MC (semidominant mutations) [3]. 

The ClC-1 protein is a homodimer with a separate ion pore within each subunit [4–7]. Voltage-dependent gating of ClC-1 has two components that are both activated by membrane depolarization: i) fast gating that occurs independently in each pore and ii) slow or common gating that operates in both pores of the dimer simultaneously [7]. Mutations causing recessive myotonia most likely affect properties of one subunit such as fast gating and/or conductance, leaving the other subunit of the heterodimer unaffected [7–10]. On the other hand mutations causing dominant myotonia affect properties of both subunits in the heterodimer and are associated with an alteration of the common gating process, which is preferentially confined to amino acids (AA) forming the dimer interface [7,11–13] but can also be affected by other regions of the structure [14–18]. In the dominant disease, a mutant monomer affects interaction with a wild type monomer subunit by exerting a dominant-negative effect, typically seen as a larger than expected shift in the voltage dependence of common gating towards more positive potentials when functional analyses of wild type-mutant heterodimers are performed [19]. The varied inheritance pattern of myotonia (recessive/dominant) appears to result from differential effects of a mutation on the channel dimer [7,8] however, detailed comprehensive structural analysis of effects of mutations in the ClC-1 protein has not been carried out.

First structural insight on ClC channels came from the discovery of bacterial homologues from *Escherichia coli* and *Salmonella typhimurium* by electron microscopy [15] and X-ray crystallography [20]. The first X-ray crystallographic structure of a eukaryotic ClC protein was determined by Feng et al. [21] for the Cl^-^/H^+^ transporter from *Cyanidioschyzon merolae* (CmClC). From the X-ray crystal structures, it became clear that individual subunits of the ClC dimer consist of a transmembrane (TM) and a cytosolic cystathionine beta-synthase (CBS) domain comprising 23 alpha-helices (A-V) and 5 beta-strands. The helices of the TM domain vary in length and are oriented somewhat obliquely relative to each other and to the plane of the membrane, with many of them not spanning the entire membrane. 

Our present study addresses two points: 1) molecular genetic diagnostics of MC by analysis of the *CLCN1* gene, and 2) structural analysis of *CLCN1* missense mutations. In the first part, we analysed DNA of Czech patients with MC. In the second part, we built a homology model of the dimeric ClC-1 channel on the basis of the crystallographic structure of the CmClC transporter [21] and identified AA forming the dimer interface and those forming the Cl^-^ ion pathway. Further, in the literature we searched for mutations of those AA for which functional analyses were performed in order to assess the correlation between the localisation of a mutation and its functional impact. Subsequently, we carried out structural analysis of these mutations to elucidate their effect on the protein structure and function and simultaneously described the effect of novel missense mutations.

## Materials and Methods

### Ethics Statement

The study has been approved by the Ethical committee of University Hospital Brno and the Ethical committee of University Hospital Motol and has therefore been performed in accordance with ethical standards laid down in the 1964 Declaration of Helsinki. All participants provided their written informed consent which was approved by the committees.

### Patients

For analysis of the *CLCN1* gene, patients were sent from Departments of Neurology and Medical Genetics within the Czech Republic. Detailed clinical information (age at onset, myotonia degree, myotonia distribution, specific clinical findings, muscle hypertrophy, muscle weakness, etc.) were requested retrospectively on the basis of positive results of DNA analysis. Out of the total number of 51 patients with mutation/mutations in *CLCN1*, valid clinical information was obtained in 34 patients. 

### DNA analysis

Genomic DNA was extracted from peripheral blood leukocytes by the standard salting-out method, and amplified by PCR. Primers for amplification of all exons and adjacent intron sequences are described in File S1 as well as the conditions of particular PCRs, see Table S1 in File S1. PCR products were sequenced directly using the BigDye Terminator Cycle Sequencing Kit (Applied Biosystems) and analysed on the ABI 3130Xl Genetic Analyzer (Applied Biosystems). The resulting sequences were compared with the *CLCN1* NCBI reference sequence (NG_009815.1). To find out whether the detected sequence variations were described previously, we used literature and databases such as the Leiden Open Variation Database (LOVD, http://chromium.liacs.nl/LOVD2/home.php?action=switch_db) and the Human Gene Mutation Database (HGMD, http://www.hgmd.cf.ac.uk/ac/index.php). All novel missense mutations were screened in a control panel consisting of DNA from 200 healthy Czech individuals. For prediction of effects of mutations on splicing of pre-mRNA, the *in silico* tools NetGene2 (http://www.cbs.dtu.dk/services/NetGene2/) and SpliceView (http://zeus2.itb.cnr.it/~webgene/wwwspliceview.html) were used. The nomenclature of mutations was implemented according to the current HGVS recommendations (http://www.hgvs.org/mutnomen/). In patients with one mutation in the *CLCN1* gene, detection of deletions/duplications was performed using Multiplex ligation-dependent probe amplification (MLPA). We used the SALSA MLPA kit P350-B1 CLCN1-KCNJ2 according to the manufacturer’s guideline (MRC Holland). This kit contains probes and primers for all 23 *CLCN1* exons.

### Homology modelling of the human ClC-1 dimer and structural analysis of the CLCN1 mutations

The crystallographic structure of the CmClC transporter from *Cyanidioschyzon merolae* (PDB code 3org, chain A, [21]) was used as a template for the human ClC-1 protein (we modeled the TM domain, AA 120 to 593, using the I-TASSER server [22]). Regions 319-347, 408-420, and 430-460 which are disordered in the initial structure were not considered in detailed structural analyses. The human dimeric ClC-1 structure was obtained by superimposing the homology model of the human monomers and the dimeric CmClC crystal structure (PDB entry 3org, chains A and D) using the program VMD http://www.ks.uiuc.edu/Research/vmd/ [23]. The chloride pathway inside the ClC-1 model was identified using the software Mole*online* 2.0 http://mole.upol.cz/ [24] and the dimer interface by the program PyMOL (http://www.pymol.org, The PyMOL Molecular Graphics System, Version 1.3 Schrödinger, LLC). Structural figures were generated by PyMOL and VMD.

As in our previous study [25], we evaluated the impact of selected *CLCN1* missense mutations on the protein structure and function using *in silico* tools. In particular, we detected specific side chain contacts (H-bonds, aromatic interactions) of AA in the 3D structure of the wild type protein based on visual inspection using the VMD program. Loss of these contacts upon a mutation often results in destabilization of the protein structure. In addition, we measured the buriedness of wild type AA corresponding to a relative accessible surface area (RSA) of <15 % [26]. Replacement of buried AA is more likely to be associated with structural defects especially when volume, charge and polarity change upon a mutation, and thus for buried residues we measured these parameters. Volume change upon mutation was calculated according to [27], and a change with an absolute value of ≥30 Å^3^ was considered destabilizing [28]. A charge change upon mutation was considered between charged and uncharged AA and a polarity change was considered between nonpolar (Leu, Ile, Phe, Trp, Cys, Met, Val), polar (Tyr, Pro, Ala, Thr, Gly, Ser), and very polar (His, Arg, Gln, Lys, Asn, Glu, Asp) AA [29].

## Results

### DNA analysis of the *CLCN1* gene

In this set of probands with a diagnosis of MC, we have 6 patients with one mutation detected in the *CLCN1* gene (Table 1). The mutation p.(Trp164Arg) identified in patient 1 is associated with dominant inheritance (the patient´s mother carrying the mutation also has MC). In another study [30], p.(Trp164Arg) was described in a patient with the genotype [p.(Trp164Arg)];[c.1471+1GA] but no information concerning MC in family members was included. The mutation p.(Met560Thr) was found in patient 2, whose parents died many years ago and probably were without any symptoms of myotonia. This mutation was also described in a patient with typical myotonia (genotype p.[Met560Thr];[Tyr261Cys]) and in his father with mild symptoms of myotonia (genotype p.[Met560Thr];[=]) [31]. The most frequent mutation among our MC probands is the semidominant mutation p.(Arg894*) which was detected in 38 disease alleles (39.6 %). This mutation can be expressed by the dominant or the recessive manner of inheritance. In a previous study [32], this mutation was dominant with late onset of symptoms (22-30 years), dominant with early onset of symptoms (7 years), or recessive. In patients 3-6, the mutation p.(Arg894*) was present as one mutation detected in the *CLCN1* gene; the patients´ parents were without clinical symptoms of MC, but detailed neurological examination was performed only in the parents of patient 6. 

**Table 1:** *CLCN1* mutations detected in Czech MC patients.

No. of patient	Phenotype	1^st^ Mutation (cDNA and protein level)	2^nd^ Mutation (cDNA and protein level)	3^rd^ Mutation (cDNA and protein level)
1	TD	c.490T>C, p.(Trp164Arg)		
2	MC, isolated occurrence in family	c.1679T>C, p.(Met560Thr)		
3	MC, isolated occurrence in family	c.2680C>T, p.(Arg894*)		
4	MC, isolated occurrence in family	c.2680C>T, p.(Arg894*)		
5	MC, isolated occurrence in family	c.2680C>T, p.(Arg894*)		
6	MC, isolated occurrence in family	c.2680C>T, p.(Arg894*)		
7	BD	c.32delG, p.(Gly11Valfs*66)	c.2680C>T, p.(Arg894*)	
8	BD	c.86A>C, p.(His29Pro)	**c.771T>A, p**.(Tyr257*)	c.1697C>T, p.(Ala566Val)
9	BD	c.180+3A>T, splicing effect	c.220C>T, p.(Gln74*)	
10	BD	c.220C>T, p.(Gln74*)	**c.587delC, p.**(Thr196Leufs*8)	
11	BD	c.220C>T, p.(Gln74*)	c.1238T>G, p.(Phe413Cys)	
12	BD	c.313C>T, p.(Arg105Cys)	c.501C>G, p.(Phe167Leu)	c.1437_1450del, p.(Pro480Hisfs*24)
13	BD	**c.433+3A>G, splicing effect**	c.1437_1450del, p.(Pro480Hisfs*24)	
14	BD	c.568_569delinsTC, p.(Gly190Ser)	c.1437_1450del, p.(Pro480Hisfs*24)	
15	BD	c.568G>A, p.(Gly190Arg)	c.2680C>T, p.(Arg894*)	
16	BD	c.568G>A, p.(Gly190Arg)	c.2680C>T, p.(Arg894*)	
17	BD	c.803C>T, p.(Thr268Met)	c.1437_1450del, p.(Pro480Hisfs*24)	
18	BD	c.870C>G, p.(Ile290Met)	c.2680C>T, p.(Arg894*)	
19	BD	**c.871G>C, p.(Glu291Gln)**	**c.1478C>T, p.(Ala493Val)**	
20	BD	**c.905A>G, p.(Tyr302Cys)**	**c.1295C>G, p.(Thr432Arg)**	c.1437_1450del, p.(Pro480Hisfs*24)
21	BD	**c.905A>G, p.(Tyr302Cys)**	**c.1295C>G, p.(Thr432Arg)**	c.2680CT, p.Arg894*
22	BD	c.908G>A, p.(Trp303*)	c.1437_1450del, p.(Pro480Hisfs*24)	
23	BD	c.1044-1156del, p.(Ala350Serfs*65)	c.2284+5C>T, splicing effect	
24	BD	c.1238T>G, p.(Phe413Cys)	c.1437_1450del, p.(Pro480Hisfs*24)	
25	BD	c.1238T>G, p.(Phe413Cys)	c.2680C>T, p.(Arg894*)	
26	BD	c.1238T>G, p.(Phe413Cys)	c.2680C>T, p.(Arg894*)	
27	BD	c.1238T>G, p.(Phe413Cys)	c.2680C>T, p.(Arg894*)	
28	BD	c.1238T>G, p.(Phe413Cys)	c.2680C>T, p.(Arg894*)	
29	BD	**c.1324_1325delAG, p.**(Ser442Profs*66)	**c.1324_1325delAG p.**(Ser442Profs*66)	
30	BD	**c.1363A>T, p.(Asn455Tyr)**	**c.1401+3A>T, splicing effect**	c.2680CT, p.(Arg894*)
31	BD	c.1437_1450del, p.(Pro480Hisfs*24)	c.1437_1450del, p.(Pro480Hisfs*24)	
32	BD	c.1437_1450del, p.(Pro480Hisfs*24)	c.1437_1450del, p.(Pro480Hisfs*24)	
33	BD	c.1437_1450del, p.(Pro480Hisfs*24)	c.1437_1450del, p.(Pro480Hisfs*24)	
34	BD	c.1437_1450del, p.(Pro480Hisfs*24)	c.1453A>G, p.(Met485Val)	
35	BD	c.1437_1450del, p.(Pro480Hisfs*24)	c.2680C>T, p.(Arg894*)	
36	BD	c.1437_1450del, p.(Pro480Hisfs*24)	c.2680C>T, p.(Arg894*)	
37	BD	c.1437_1450del, p.(Pro480Hisfs*24)	c.2680C>T, p.(Arg894*)	
38	BD	c.1437_1450del, p.(Pro480Hisfs*24)	c.2680C>T, p.(Arg894*)	
39	BD	**c.1445G>A, p.(Gly482Glu)**	**c.2508+2T>A, splicing effect**	
40	BD	c.1471+1G>A, splicing effect	c.2680C>T, p.(Arg894*)	
41	BD	c.1471+1G>A, splicing effect	c.2680C>T, p.(Arg894*)	
42	BD	c.1478C>A, p.(Ala493Glu)	c.2364+2T>A, splicing effect	
43	BD	c.2680C>T, p.(Arg894*)	c.2680C>T, p.(Arg894*)	
44	BD	c.2680C>T, p.(Arg894*)	c.2680C>T, p.(Arg894*)	
45	BD	c.2680C>T, p.(Arg894*)	c.2680C>T, p.(Arg894*)	
46	BD	c.2680C>T, p.(Arg894*)	c.2680C>T, p.(Arg894*)	
47	BD	c.2680C>T, p.(Arg894*)	c.2680C>T, p.(Arg894*)	
48	BD	c.2680C>T, p.(Arg894*)	c.2680C>T, p.(Arg894*)	
49	BD	c.2680C>T, p.(Arg894*)	c.2680C>T, p.(Arg894*)	
50	BD	c.2680C>T, p.(Arg894*)	c.2680C>T, p.(Arg894*)	
51	BD	c.2680C>T, p.(Arg894*)	c.2680C>T, p.(Arg894*)	

Mutations described by bold letters are not described previously. TD: Thomsen disease; BD: Becker disease.

In 45 MC probands, two or more mutations were identified in the *CLCN1* gene (Table 1). Mutations detected in our patients and also described in the literature and/or the databases LOVD and HGMD in association with Becker disease include p.(Gln74*), p.(Arg105Cys), p.(Phe167Leu), p.(Gly190Arg), p.(Gly190Ser), p.(Trp303*), p.(Phe413Cys), p.(Met485Val), p.(Ala493Glu), p.(Arg894*), c.180+3AT, c.1437_1450del, c.1471+1GA, c.2284+5CT, and c.2364+2TA. The mutation c.1437_1450del, p.(Pro480Hisfs*24) was present in 18 disease alleles (18.8 %) and is thus the second most frequent *CLCN1* mutation detected in our probands. The mutations p.(His29Pro) and p.(Ala566Val) detected in patient 8 are also described in LOVD, but the MC type is not mentioned there and the pathogenicity is described as unknown (see below). The mutation p.(Ile290Met) was described as dominant [33–35] but in patient 18 it is present together with p.(Arg894*). Detailed neurological examination and DNA analysis were performed in the patient´s parents; the father carries p.(Arg894*) and is without MC symptoms, and the mother carries p.(Ile290Met) and has EMG-positive but clinically silent MC. In the set of our patients with Becker disease, we described 14 new mutations: 8 types are frame-shift, splicing, or nonsense [c.32delG, c.587delC, c.1044_1156del, c.1324_1325delAG, c.433+3AG, c.1401+3AT, c.2508+2TA, p.(Tyr257*)] and 6 types are missense [p.(Glu291Gln), p.(Tyr302Cys), p.(Thr432Arg), p.(Asn455Tyr), p.(Gly482Glu), p.(Ala493Val)]. Patient 39 carries two new mutations, p.(Gly482Glu) and the splicing mutation c.2508+2TA. A mutation occurring in the same codon, p.(Gly482Arg), was identified previously in a patient with Becker disease and the genotype p.[Gly482Arg];[Pro480Hisfs*24] [36]. All potential splicing mutations showed an identical effect, loss of a donor splice site, by using *in silico* tools. The presence of all novel missense mutations was tested in DNA from 200 controls and none of them were detected. 

In five probands, we detected three sequence variants in *CLCN1*. The segregation of mutations in disease alleles was determined on the basis of DNA analysis of the parents of patient 8 (genotype p.[His29Pro; Tyr257*]; [Ala566Val]), patient 20 (genotype p.[Tyr302Cys; Thr432Arg]; [Pro480Hisfs*24]), and patient 21 (genotype p.[Tyr302Cys; Thr432Arg]; [Arg894*]). The phenotypes of these patients (if available) are described in File S1, see Table S2 in File S1. The most severe phenotypes were found in patients 8 and 14. Patient 8 (female, 39 years old, genotype p.[His29Pro; Tyr257*]; [Ala566Val]) suffers from moderate myotonia, permanent limb-girdle muscle weakness, and scoliosis. Patient 14 (male, 15 years old, genotype p.[Gly190Ser]; [Pro480Hisfs*24]) has severe myotonia, kyphoscoliosis, deformities of the feet, shortening of the Achilles tendons, and masseter and limb-girdle muscle weakness.

### Structural analysis of the *CLCN1* mutations localised in the dimer interface and the Cl^-^ ion pathway

**Figure 1:**
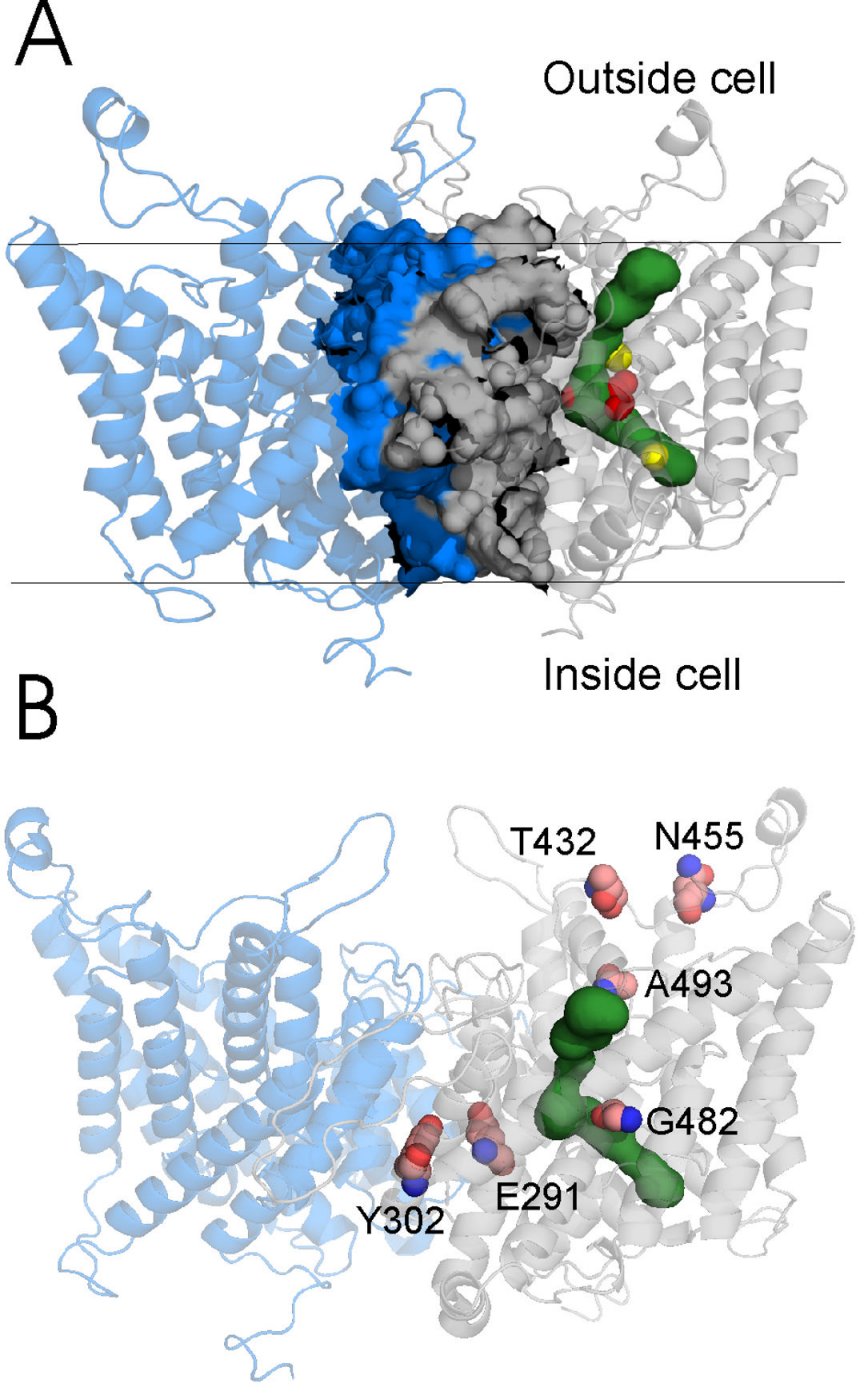
3D structure of the homology model of the human ClC-1 dimer (transmembrane domains are in blue and gray) with the Cl^-^ channel (in green) visualized in one subunit. A shows highlighted interface and the channel with the key amino acid Glu 232 (red surface) and two Cl^-^ ions (yellow balls) which were superimposed from the original X-ray structure of the CmClC transporter. B shows 6 new missense mutations mapped in one subunit.

The homology model which we built comprises AA residues 120-593 and consists of helices B-R according to the template structure (see Materials and Methods). Based on the model, we identified 40 residues forming the dimer interface and 43 residues forming the Cl^-^ ion pathway (Figure 1A and Table S3 in File S1). Residues Ser189, Glu232, and Tyr578 which are known to be essential for gating, coordination, and selectivity for Cl^−^ ions [21,37] were detected in the ion pathway (Figure 1A). The structural model shows that the central part of the Cl^-^ ion pathway comprises four glycines (residues 230, 233, 482, and 483) which probably shape this area. In addition, residues Cys278, Met485, Leu418, and Leu581 were found to be close to Cl^-^ ions superimposed from the template structure, and hence could be potentially involved in a coordination of these ions. 

Using literature data, we performed searches for mutations of AA forming the dimer interface or the Cl^-^ ion pathway in our model for which functional analysis of the wild type-mutant ClC-1 heterodimer was performed. This was done in order to assess the correlation between the localisation of a mutation and the result of functional analysis (presence/absence of a dominant-negative effect) which indicate the type of MC.

In the case of AA localised in the dimer interface (see Table S3 in File S1), 15 positions are associated with 17 mutations (Table 2) for which functional analyses were performed. Fourteen mutations showed a pronounced dominant-negative effect of the mutation in the wild type-mutant heterodimer, 2 a weak dominant-negative effect, and one mutation no dominant-negative effect (Table 2). Structural analysis revealed that most of these mutations occur in positions which are buried in the interface. The positions 317 and 552 are exceptions, localised on the protein surface pointing into extracellular space. Some substitutions with a dominant-negative effect do not exhibit a significant structural defect, e.g. p.(Ile290Met) or p.(Phe306Leu), while others show changes of volume/charge/polarity, e.g. p.(Phe297Ser) or p.(Tyr302His) (Table 2). The mutation p.(Glu291Lys) shows the most significant structural defect and no dominant-negative effect was seen in this case. This mutation exhibits loss of contacts and simultaneously changes of volume and charge. In particular, structural analysis of our ClC-1 model reveals H-bonds between the side chain of Glu291 (positioned in the helix H) and the main chains of Val540 and Ser541 (positioned in the helix P) that stabilize the arrangement between the two helices at the interface (Figure S1 in File S1). Introduction of a positively charged lysine at position 291 prevents such interactions and probably results in protein misfolding. This is in agreement with the identified recessive inheritance of this mutation [36] and with a previous experimental study [34]. In that study, a complete abolishment of channel activity was found in the homodimeric channel, while in the wild type-mutant heterodimeric channel observed currents were about 50% of wild type, consistent with the fact that p.(Glu291Lys) is inherited recessively. The position 291 is known to be associated with various effects according to the mutant AA. For example, p.(Glu291Asp) was found to have a dominant-negative effect in the wild type-mutant ClC-1 heterodimer (Table 2) probably due to conservation of negative charge and partial compensation of the contacts due to the similarity between glutamate and aspartate [34].

**Table 2:** Previously identified *CLCN1* mutations associated with AA localised directly on the dimer interface or along the Cl^-^ ion pathway in the dimeric ClC-1 model with functional analysis of the wild type-mutant heterodimer (see also AA in bold letters in Table S3 in File S1).

**Mutation/Position in the secondary structure**	**Result of functional analysis, effect of mutation on wt-mut^$^ heterodimer**	**Side chain interactions of AA in the starting structure**	**Buriedness of AA (RSA in %)^&^**	**Change of Volume (Å^3^)^@^/Charge/Polarity/ upon mutation**
**Mutations localized in the dimer interface**				
p.(Leu283Phe)/α-helix H	Dominant-negative effect [16]	No	7	+23/No/No
p.(Ile290Met)/α-helix H	Dominant-negative effect [7,34,38]	No	7	-4/No/No
p.(Glu291Lys)/α-helix H	Protein misfolding and protein degradation [34]	H-bonds: Glu(OE1)-Ser541(N), Glu(OE2)-Val540(N)	9	+30/Yes/No
p.(Glu291Asp)/α-helix H	Dominant-negative effect [34]	H-bonds: Glu(OE1)-Ser541(N), Glu(OE2)-Val540(N)	9	-27/No/No
p.(Phe297Ser/β-strand 1	Dominant-negative effect [39]	No	9	-101/No/Yes
p.(Tyr302His)/α-helix I	Dominant-negative effect [40]	No	9	-35/Yes/Yes
c.(Trp303Arg)/α-helix I	Dominant-negative effect [39]	Clashes^%^	8	-54/Yes/Yes
p.(Phe306Leu)/α-helix I	Dominant-negative effect [39]	No	2	-23/No/No
p.(Phe307Ser)/α-helix I	Dominant-negative effect [8,39]	No	13	-101/No/Yes
p.(Thr310Met)/α-helix I	Dominant-negative effect [16]	No	7	+47/No/Yes
p.(Ala313Thr)/α-helix I	Dominant-negative effect [8,39]	No	4	+28/No/No
p.(Ala313Val)/α-helix I	Dominant-negative effect [39]	No	4	+51/No/Yes
p.(Arg317Gln)/α-helix I	Dominant-negative effect [34]	No	59	-
p.(Gln552Arg)/turn	Dominant-negative effect [34]	No	40	-
p.(Ile553Phe)/turn	Weak dominant-negative effect [41]	No	0	+23/No/No
p.(His555Asn)/turn	Dominant-negative effect [41]	No	16	-
p.(Ile556Asn)/α-helix Q	Weak dominant-negative effect [7,8]	No	0	-53/Yes/Yes
**Mutations localized along the Cl- pathway**				
p.(Gly230Glu)/turn	Weak dominant-negative effect, mutation does not shift the ClC-1 voltage dependence to positive voltages (as in fully dominant mutations) [36,42,43,44]	No	0	+78/Yes/Yes
p.(Gly233Ser)/α-helix F	No dominant-negative effect [45]	No	0	+29/No/No
p.(Arg421Cys)/α-helix L	Weak dominant-negative effect [40]	H-bond: Arg(NH2)-Phe279(O)	29	-
p.(Phe428Ser)/α-helix L	No dominant-negative effect [16]	Interaction of aromatic rings: Phe428-Phe351	20	-
p.(Met485Val)/α-helix N	No dominant-negative effect [8,9,36]	No	10	+23/No/No
p.(Thr550Met)/turn	Dominant negative effect [16]	No	47	-

^$^ Wt-mut refers to wild type-mutant. ^&^ AA were considered buried if RSA < 15%. ^@^ Volume change with an absolute value of ≥ 30 Å^3^ was considered destabilizing. ^%^ There are close contacts in this area so it is difficult to detect contacts for this residue

In the case of AA localised in the Cl^-^ ion pathway (Table S3 in File S1), 6 mutations out of 43 were found where functional analysis of the wild-type-mutant heterodimer was performed (Table 2). Five mutations have no pronounced dominant-negative effect in the wild type-mutant heterodimer; two of these are substitutions of glycine residues (positions 230 and 233 mentioned above) of which the first is part of a turn structure while the second one is positioned in an α-helix, typical for transmembrane helices where glycines take part in helix-helix interactions [46]. Substitutions of both glycines are more likely to change the local architecture or even cause misfolding [44,45]. Two other mutations are positioned in the external mouth of the channel (positions 421 and 428) and have single contacts in the starting model (Table 2) which are abolished upon mutation. The mutation p.(Met485Val) does not show significant structural defects (Table 2), but as mentioned above residue 485 probably coordinates Cl^-^ ions in the channel, hence p.(Met485Val) might directly impact the channel activity. The mutation p.(Thr550Met) with a dominant-negative effect is positioned in the external mouth of the channel very close to the dimer interface and does not show a significant structural defect (Table 2).

### Structural analysis of the novel MC missense mutations

Six novel *CLCN1* missense mutations were identified in our patients (Tables 1, Table 3, and Figure 1B), all associated with recessive MC. The first, p.(Glu291Gln), occurs at AA position 291 (in the dimer interface, see above). This replacement of glutamate with uncharged glutamine most likely results in protein misfolding similarly to p.(Glu291Lys) which agrees with the observed recessive inheritance (Table 1). The mutations p.(Ala493Val) and p.(Gly482Glu) are positioned close to and in the Cl^-^ ion pathway, respectively, and the first will probably lead to a local structural defect (most likely affecting only the mutant subunit in the wild type-mutant heterodimer) while the second may induce larger structural perturbation due to replacement of the glycine residue in the turn structure with larger charged glutamate (which may lead to misfolding). Both these structural defects are in agreement with the observed recessive phenotype. Two other mutations [p.(Thr432Arg) and p.(Asn455Tyr)] are positioned on the surface of the protein (pointing into extracellular space) in the area which is disordered in the native structure. Therefore, we did not determine their structural impact but considering their positions on the exterior surface they will most likely cause only local structural changes, if any. The mutation p.(Thr432Arg) was identified in patients 20 and 21 on one allele together with another new mutation p.(Tyr302Cys) which occurs on the dimer interface and exhibits a change of volume and polarity (Table 3). Such defect was detected in mutations associated with dominant MC localized at the dimer interface (Table 2). In addition, AA position 302 is associated with mutation p.(Tyr302His) (Table 2) that shows a dominant-negative effect in the wild-type mutant heterodimer [40]. The dominant negative effect of p.(Tyr302His) would explain EMG myotonic “runs” shown by an individual carrying this mutation, but it is obviously not sufficient to cause MC clinical symptoms when present alone in heterozygous state [40]. In our patients 20 and 21, the inheritance pattern of MC corresponds with the recessive type however the detailed neurological examination of their parents was not performed. 

**Table 3:** Structural analysis of newly identified *CLCN1* missense mutations.

**Mutation/position in the secondary structure**	**Localization in the protein**	**Side chain interactions in the starting structure**	**Buriedness of AA (RSA in %)^&^**	**Change of Volume (Å^3^)^@^/charge/polarity/ upon mutation**
p.(Glu291Gln)/α-helix H	Dimer interface	H-bonds: Glu(OE1)-Ser541(N), Glu(OE2)-Val540(N)	9	5/Yes/No
p.(Tyr302Cys)/α-helix I	Dimer interface	No	9	-85/No/Yes
p.(Thr432Arg)^*^/turn	On the surface	-	-	-
p.(Asn455Tyr)^*^/turn	On the surface	-	-	-
p.(Gly482Glu)/turn	Channel	No	7	+78/Yes/Yes
p.(Ala493Val)/α-helix N	Inside, but outside the channel and dimer interface	No	1	+51/No/Yes

^&^ AA were considered buried if RSA < 15%. ^@^ Volume change with an absolute value of ≥ 30 Å^3^ was considered destabilizing. ^*^ This residue is positioned on the surface in extracellular space in the region which is disordered in the X-ray structure. Thus, detailed structural description was not performed.

## Discussion

In this study, we present results of sequence analysis of the *CLCN1* gene performed in Czech patients with myotonia congenita. Mutations associated with the disease were identified in 51 probands; 14 mutations are new, 8 types are frame-shift, splicing, or nonsense and 6 are missense (Table 1). In the cohort of our patients, two mutations p.(Arg894*) and c.1437_1450del are significantly represented and account for 39.6% and 18.8% of mutant MC alleles, respectively. The third most frequent mutation is p.(Phe413Cys), detected in 6% of the disease alleles. This is in line with data for other European countries (Table S4 in File S1). In particular, all three mutations are the most frequently detected mutations in United Kingdom [39]. The mutation p.(Arg894*) is the most frequently observed mutation in Russia [47], Northern Scandinavia [48], and Denmark [32], but it is also significantly represented in Netherlands [49], Spain [40] and Italy [50]. The p.(Phe413Cys) belongs to one of the three most frequent mutations in Northern Scandinavia and Netherlands and the c.1437_1450del mutation to one of the three most frequent mutations in Russia and Denmark.

Further, we created a homology model of the human dimeric ClC-1 channel (Figure 1A) based on the crystallographic structure of the CmClC transporter which has been shown to share many common features with the other members of the ClC family [21]. Using our *in silico* ClC-1 model, we mapped the new missense mutations onto the protein structure (Figure 1B) and analyzed their impacts. Further, based on our 3D model, we identified AA forming the dimer interface and those forming the Cl^-^ ion pathway (Figure 1A and Table S3 in File S1). In the next step, we performed searches for mutations of AA forming the dimer interface or the Cl^-^ ion pathway in our model for which functional analysis of the wild type-mutant ClC-1 heterodimer was performed. This was done in order to assess the correlation between the localisation of a mutation and the result of functional analysis (presence/absence of a dominant-negative effect) which indicate the type of MC. We did not consider clinical data for this correlation as they are often not complete and thus less reliable than the functional analysis.

Out of 40 residues forming the dimer interface, 15 positions were associated with 17 missense mutations for which functional analysis was performed (Table 2). As expected, these mutations were mainly associated with a dominant-negative effect. Structural analysis of the mutations in the homology model revealed that some mutations associated with a dominant-negative effect exhibit change of volume, charge and/or polarity while others do not show significant structural defects (Table 2). This indicates that even relatively conserved substitution at the interface impacts the dimeric structure and results in a dominant-negative effect, in accord with the fact that the dimer interface has a high shape complementarity index (similar to an antibody-antigen interface) [21] leading to low tolerance of substitutions in this area. The only exception considering a dominant-negative effect in the interface was the mutation p.(Glu291Lys) associated with the recessive type of MC. Structural analysis of our homology model showed that this mutation impacts protein structure significantly introducing combined effect (loss of contacts and change of volume and charge) which probably results in protein misfolding in agreement with an experimental study [34]. Mutations leading to a dominant-negative effect can however be found at various positions in the ClC-1 protein and are not associated only with the dimer interface [9,16]. These mutations probably induce rearrangements that propagate through the protein structure and affect the second subunit. Another example is a mutation with a dominant-negative effect p.(Thr550Met) (Table 2) positioned in the external mouth of the Cl^-^ ion pathway very close to the dimer interface in our model structure; even though this residue does not form part of the interface its proximity might be responsible for the observed dominant-negative effect. Mutations localized inside the Cl^-^ ion pathway were found to have no dominant-negative effect in the wild type-mutant heterodimer (Table 2). The *in silico* analysis suggested that these mutations can impose various defects, e.g. loss of ability to bind Cl^-^ ions or various structural disruptions.

## Conclusions

In this set of Czech probands with MC, 51 were found with mutation(s) in the *CLCN1* gene, 14 of which were new. In the homology model of the human dimeric ClC-1 channel coded by the *CLCN1* gene, mutations of AA forming the dimer interface are prevalently associated with a dominant-negative effect and dominant inheritance, even though mutations leading to no dominant-negative effect and recessive inheritance can also be found there. We show that dominant/recessive mutations in this area differ in their impact on the protein structure. On the contrary, mutations of AA localized inside the Cl^-^ ion pathway were found to have no pronounced dominant negative effect. Structural analysis revealed that these mutations can directly affect the channel activity or the local structure of one subunit, or induce misfolding of the mutant subunit. Our results demonstrate structure-function relationships in the ClC-1 protein which are relevant to understanding the molecular pathogenesis of MC.

## Supporting Information

File S1 **Contains Table S1, S2, S3, S4 and Figure S1.**
(DOC)
