# Supplementary material for: CLCN1 Mutations in Czech Patients with Myotonia Congenita, In Silico Analysis of Novel and Known Mutations in the Human Dimeric Skeletal Muscle Chloride Channel
Source: PLoS One. 2013 Dec 11;8(12):e82549. doi: 10.1371/journal.pone.0082549 (PMC3859631; doi:10.1371/journal.pone.0082549)
Supplement: File S1 — Contains Table S1, S2, S3, S4 and Figure S1. (DOC) [file pone.0082549.s001.doc]

# Supporting information

**Table S1.** Primers used for amplification of the *CLCN1* exons and adjacent intron regions

| Gene | Exon | Sequence of primers (5´→ 3´direction) | Length of PCR product (bp) | Conditions (program PCR, T_a_) |
| --- | --- | --- | --- | --- |
| *CLCN1* | 1 | Forward: CACTGCACTCACACCTGCTT Reverse: CACTGCTCCCTCTTTTCACC | 486 | PCR2, 65°C |
| *CLCN1* | 2 | Forward: GGGATGACCACAAAGTCACC Reverse: TACGTACGTGCAATGGGATG | 360 | PCR1, 65°C |
| *CLCN1* | 3 | Forward: TCGTTAGCTGCTTTTCTCTCTC Reverse: CCCTGCTTAACATTCGCTTC | 349 | PCR1, 60°C |
| *CLCN1* | 4+5 | Forward: CTCAGAAGGGGCACACAGA Reverse: GATCCACTTCCACTCCCAGA | 696 | PCR2, 62°C |
| *CLCN1* | 6 | Forward: CACAGTGCCTGGAGTAAGGAA Reverse: AACACCCTGGACACACAGC | 244 | PCR1, 65°C |
| *CLCN1* | 7 | Forward: CTTATTCCCCATCCCTGCTT Reverse: ACCAGCACTTGTGGATACTGATT | 251 | PCR1, 60°C |
| *CLCN1* | 8+9+10 | Forward: CTGCTTCCACCCAGATTCAT Reverse: CATTTAATGCCTTCCCCAAA | 1087 | PCR2, 60°C |
| *CLCN1* | 11+12 | Forward: ACTTCAGCTTGCCATCGTTC Reverse: CCTTCCTACCCTATGCAAACC | 616 | PCR2, 60°C |
| *CLCN1* | 13+14 | Forward: AGTGGGAAGGGAATTGTGTG Reverse: CCGTATTGTGTAGCCTAGAATGAA | 545 | PCR2, 62°C |
| *CLCN1* | 15+16 | Forward: CGTGTTATTCCCATCCCATC Reverse: ATGGCCCCTAAAGACAAAGC | 765 | PCR2, 62°C |
| *CLCN1* | 17 | Forward: GCCTCTCCTGTTCCTTCTCA Reverse: TCAGGGAAACTACGCCTGTC | 436 | PCR2, 60°C |
| *CLCN1* | 18+19+20 | Forward: GTTCGCTTTCCCAGAACATC Reverse: ACACTTCCCATCCAGACCAC | 1024 | PCR2, 65°C |
| *CLCN1* | 21+22 | Forward: GTTCTTGCATGTTCCCAGATT Reverse: TGAGGGGACTTCTGAGATGC | 506 | PCR2, 60°C |
| *CLCN1* | 23 | Forward: TCCTTTCATTGTACCTGTTCTTTTC Reverse: ACGACATTGGCATGACCTC | 578 | PCR2, 62°C |

T_a_: annealing temperature. Amplification conditions: Reaction mixtures with a final volume of 25 μl contained 200 ng DNA, 1x Taq Buffer with KCl (Fermentas), 0.2 mM dNTPs, 1.5 mM MgCl_2_, 0.5 μM each PCR primer, and 1 U Taq DNA Polymerase (Fermentas). Amplifications were performed in the following cycling conditions: 94 °C for 5 min, followed by 30 cycles at 94°C for 30 sec, T_a_°C for 30 sec, and 72 °C for 30 sec (PCR1 program); 94 °C for 5 min, followed by 30 cycles at 94 °C for 50 sec, T_a_ for 1 min, and 72 °C for 1 min (PCR2 program).

**Table S2.** Clinical findings in Czech MC patients with *CLCN1* mutations

| No./ sex | Mutation | Year of birth | Age at presentation (years) | Age at onset (years) | Myotonia degree | Grip/ percussion myotonia | Myotonia distribution | Specific clinical findings | Muscle hypertrophy | Transient muscle weakness | Worsening in cold | Warm up | EMG | CK (μkat/l) | MC in parents |
| --- | --- | --- | --- | --- | --- | --- | --- | --- | --- | --- | --- | --- | --- | --- | --- |
| 1/M | p.(Trp164Arg) | 1984 | 27 | 10 | Moderate | Yes/yes | LL>UL | No | LL>UL | No | Yes | Yes | + | 11.40 | Mother with the mutation has MC. |
| 2/M | p.(Met560Thr) | 1953 | 57 | 6 | Moderate | Yes/no | LL=F>UL | No | No | No | Yes | Yes | + | NM | No |
| 3/F | p.(Arg894*) | 1966 | 46 | 10 | Mild | Yes/yes | LL=UL=F | No | No | No | Yes | Yes | + | 6.00 | No |
| 4/F | p.(Arg894*) | 1965 | 46 | 35 | Moderate | Yes/no | LL=UL | No | No | No | Yes | Yes | + | NM | No |
| 6/F | p.(Arg894*) | 1976 | 35 | 25 | Mild | Yes/no | LL=UL=F | No | No | No | Yes | Yes | + | 3.58 | Mother with the mutation is clinically without MC, pronounced EMG myopathy was detected. |
| 7/F | p.(Gly11Valfs*66)/p.(Arg894*) | 1971 | 40 | 6 | Severe (probably overlapping with somatoform disorder) | Yes/no | LL>UL>F | No | No | No | Yes | Yes | + | NM | No |
| 8/F | p.(His29Pro)/ p.(Tyr257*)/ p.(Ala566Val) | 1972 | 39 | 6 | Moderate | Yes/no | LL=UL>F | Permanent limb-girdle muscle weakness,scoliosis | Generalised | No | Yes | Yes | + | 2.57 | No |
| 9/M | c.180+3A>T/ p.(Gln74*) | 1977 | 33 | 4 | Severe | Yes/yes | LL=UL=F | Pectus excavatum | Generalised | No | Yes | Yes | + | 12.77 | No |
| 10/F | p.(Gln74*)/ p.(Thr196Leufs*8) | 1975 | 35 | 4 | Mild | Yes/no | LL=UL | No | No | No | Yes | Yes | + | 2.47 | No |
| 11/F | p.(Gln74*)/ p.(Phe413Cys) | 1944 | 56 | 6 | Moderate | Yes/yes | LL>UL>F | No | Quadriceps | No | Yes | Yes | + | 3.57 | No |
| 13/M | c.433+3A>G/ p.(Pro480Hisfs*24) | 1986 | 25 | 6 | Moderate | NM | LL>UL | No | LL | No | Yes | Yes | + | NM | No |
| 14/M | p.(Gly190Ser)/ p.(Pro480Hisfs*24) | 1996 | 15 | 1 | Severe | NM | LL=UL=F | Rigid walking (only several meters), kyfoscoliosis, masseter and limb-girdle muscle weakness, Achilles tendon contractures, feet deformities | NM | UL | Yes | Yes | + | NM | No |
| 15/M | p.(Gly190Arg)/ p.(Arg894*) | 1961 | 50 | 6 | Moderate | Yes/yes | LL=UL>F | No | Generalised | No | Yes | Yes | + | 5.38 | No |
| 16/F | p.(Gly190Arg)/ p.(Arg894*) | 1985 | 26 | 4 | Moderate | Yes/yes | LL>UL=F | No | Generalised | No | No | Yes | + | 1.08 | No |
| 17/F | p.(Thr268Met)/ p.(Pro480Hisfs*24) | 2008 | 3 | 3 | Moderate | Yes/yes | LL=UL | No | LL=UL | LL | No | Yes | + | 4.2 | Father has MC symptoms, no DNA analysis and detailed neurological examination were performed. |
| 18/F | p.(Ile290Met)/ p.(Arg894*) | 1981 | 28 | 6 | Very severe | Yes/yes | UL>LL=F | No | Generalised | No | Yes | Yes | + | 7.08 | Mother carrying p.(Ile290Met) has EMG- detected but clinically silent MC. |
| 19/M | p.(Glu291Gln)/ p.(Ala493Val) | 1992 | 10 | 3 | Moderate | Yes/yes | LL=UL>F | No | LL=UL | Yes | Yes | Yes | + | NM | No |
| 21/M | p.(Tyr302Cys)/ p.(Thr432Arg) | 1978 | 32 | 5 | Moderate | NM | LL=UL>F | Lid myotonia | Generalised | UL | Yes | Yes | + | 1.94 | No |
| 22/F | p.(Trp303*)/ p.(Pro480Hisfs*24) | 2004 | 5 | 2 | Moderate | NM | LL | No | Generalised | No | No | Yes | + | 1.83 | No |
| 23/F | p.(Ala350Serfs*65)/ c.2284+5C>T | 1990 | 21 | 10 | Mild | Yes/no | LL=UL>F | No | LL>UL | Generalised | Yes | Yes | + | 5.66 | No |
| 24/M | p.(Phe413Cys)/ p.(Pro480Hisfs*24) | 1996 | 16 | 3 | Severe | Yes/yes | LL>UL=F | No | Generalised | No | Yes | Yes | + | 3.57 | No |
| 25/F | p.(Phe413Cys)/ p.(Arg894*) | 1971 | 37 | 3 | Moderate | Yes/yes | LL>UL=F | No | Quadriceps | No | Yes | Yes | + | NM | No |
| 29/M | p.(Ser442Profs*66)/ p.(Ser442Profs*66) | 1986 | 22 | 14 | Mild | Yes/yes | LL=UL=F | No | No | No | No | Yes | + | NM | No |
| 31/F | p.(Pro480Hisfs*24)/ p.(Pro480Hisfs*24) | 1987 | 22 | 4 | Moderate | Yes/no | LL>UL=F | Scoliosis, Achilles tendon contractures | Quadriceps, calves | Generalised | Yes | Yes | + | 5.65 | No (Mother and father are cousins.) |
| 32/F | p.(Pro480Hisfs*24)/ p.(Pro480Hisfs*24) | 1989 | 20 | 4 | Moderate | Yes/no | LL=UL | No | LL | LL | No | Yes | + | 2.29 | No |
| 34/M | p.(Pro480Hisfs*24)/ p.(Pro480Hisfs*24) | 1992 | 17 | 15 | Moderate | Not mentioned | LL>UL | No | LL | LL | No | Yes | + | NM | No |
| 35/M | p.(Pro480Hisfs*24)/ p.(Arg894*) | 1965 | 44 | 3 | Mild | Yes/no | UL=LL>F | No | Generalised | No | Yes | Yes | NM | 1.21 | No |
| 36/M | p.(Pro480Hisfs*24)/ p.(Arg894*) | 1989 | 21 | 10 | Moderate | Yes/no | LL>UL>F | No | Generalised | No | Yes | yes | + | 4.98 | No |
| 37/M | p.(Pro480Hisfs*24)/ p.(Arg894*) | 1996 | 16 | 12 | Moderate | Yes/no | LL>UL=F | No | Generalised | No | No | Yes | + | NM | No |
| 41/F | c.1471+1G>A/ p.(Arg894*) | 1972 | 39 | 10 | Mild | Yes/no | LL=UL | No | LL | No | Yes | Yes | + | 1.81 | No |
| 42/F | p.(Ala493Glu)/ c.2364+2T>A | 2000 | 12 | 5 | Moderate | Yes/no | LL>UL=F | No | Generalised | No | No | Yes | + | NM | No |
| 49/F | p.(Arg894*)/ p.(Arg894*) | 1969 | 41 | 30 | Mild | Yes/no | UL | No | No | No | No | No | + | 1.61 | No |
| 50/F | p.(Arg894*)/ p.(Arg894*) | 1969 | 42 | 6 | Mild | Yes/yes | LL=UL=F | No | No | Generalised | Yes | Yes | + | 3.22 | Mother carrying p.(Arg894*) has MC symptoms. |
| 51/F | p.(Arg894*)/ p.(Arg894*) | 1956 | 50 | 10 | Moderate | Yes/no | LL>UL=F | No | LL | No | Yes | Yes | + | 2.82 | No |

M: male; F: female; NM: not mentioned; LL: lower limb; UL: upper limb; F: face; +: myotonic discharges, CK: creatine kinase. In patients describing no family history of disease, detailed neurological examination of parents was not performed.

**Table S3.** Amino acids directly forming the dimer interface and the Cl^-^ ion pathway in the dimeric ClC-1 model

| Region of ClC1 | AA directly forming the selected region |
| --- | --- |
| Dimer interface | **283**, 287, **290**, **291**, 292, 294, 295, 296, **297**, 298, 299, 300, **302**, **303,** **306,** **307**, **310**, 311, **313**, 314, **317**, 538, 540, 544, 548, **552**, **553**, **555**, **556**, 557, 560, 561, 564, 565, 567, 568, 569, 571, 572, 573 |
| Cl^-^ ion pathway | 187, 188, 189, 190, 191, 192, 228, 229, **230**, 232, **233**, 279, 278, 373, 418, **421**, 424, 425, **428**, 474, 475, 476, 478, 481, 482, 483, 484, **485**, 486, 487, 488, 526, 536, 537, 546, 547, 549, **550**, 551, 577, 578, 581, 585 |

AA, in which functional analysis of wild type-mutant heterodimer was performed, are in bold letters

**Table S4. Occurrence of the most frequent *CLCN1* mutations in the Czech Republic and other European countries**

| **Country** | **No. of probands/disease alleles** | **The most frequent mutations** | **No. of probands with the mutation** | **No. of alleles with the mutation** |
| --- | --- | --- | --- | --- |
| Czech Republic (this study) | 51/96 | **p.(Arg894*)** | 29 (56.9 %) | 38 (37.5 %) |
|  |  | **c.1437_1450del** | 15 (29.4 %) | 18 (18.8 %) |
|  |  | **p.(Phe413Cys)** | 6 (11.8 %) | 6 (6.3 %) |
| Italy [1] | 93/not mentioned | p.(Phe167Leu) | 13 (14 %) | Not mentioned |
|  |  | p.(Gly190Ser) | 12 (13 %) | Not mentioned |
|  |  | c.180+3A>T | 11 (12 %) | Not mentioned |
| Spain [2] | 32/60 | c.180+3A>T | 16 (50 %) | 18 (30 %) |
|  |  | p.(Met485Val) | 6 (18.8 %) | 8 (13.3 %) |
|  |  | p.(Ala167Leu) | 4 (12.5 %) | 4 (6.7 %) |
| UK [3] | 22/30 | **c.1437_1450del** | 3 (13.6 %) | 4 (13.3 %) |
|  |  | **p.(Arg894*)** | 2^$^ (9 %) | 2 (6.7 %) |
|  |  | **p.(Phe413Cys)** | 2^$^ (9 %) | 2 (6.7 %) |
| Russia [4] | 66/118 | **p.(Arg894*)** | 29 (43.9 %) | 36 (30.5 %) |
|  |  | **c.1437_1450del** | 11 (16.7 %) | 11 (9.3 %) |
|  |  | p.(Gly190Ser) | 7 (10.6 %) | 7 (5.9 %) |
| Northern Scandinavia [5] | 16/32 | **p.(Arg894*)** | 10 (62.5 %) | 13 (40.6 %) |
|  |  | **p.(Phe413Cys)** | 7 (43.8 %) | 8 (25 %) |
|  |  | p.(Ala531Val) | 5 (31.3 % ) | 5 (15.6 %) |
| Denmark [6] | 8/12 | **p.(Arg894*)** | 4 (50 %) | 4 (33.3 %) |
|  |  | p.(Pro480Leu) | 2 (25 %) | 2 (16.7 %) |
|  |  | **c.1437_1450del** | 1^$^ (12.5%) | 1(8.3%) |
| Netherlands [7] | 32/56 | **p.(Phe413Cys)** | 8 (23 %) | 9 (16.1 %) |
|  |  | p.(Gly285Glu) | 5 (15.6 %) | 7 (12.5 %) |
|  |  | **p.(Arg894*)** | 3 (9.4 %) | 5 (8.9 %) |

^$^ Other mutations with comparable frequency occur in the original study, but they are not listed in this table as they are not relevant to frequent mutations in the Czech Republic.

**
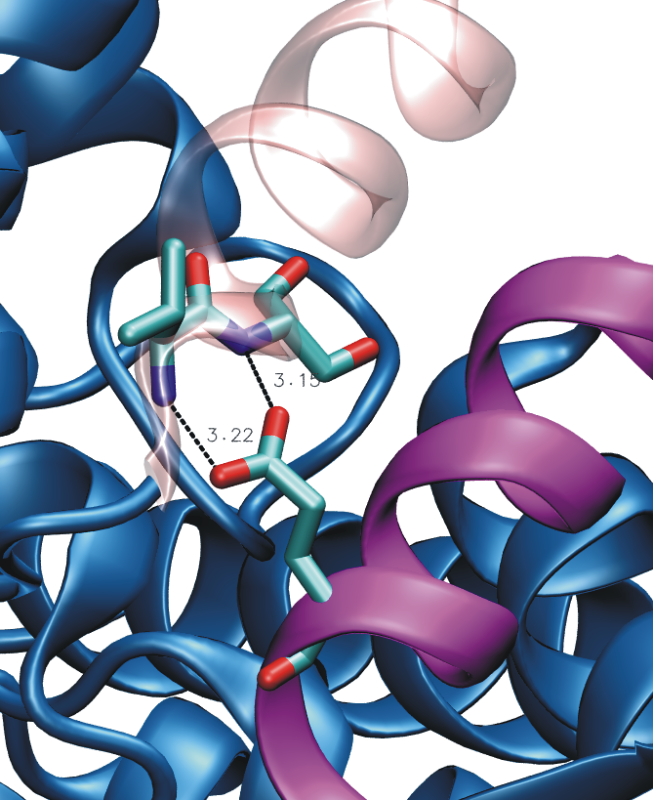
**

**Figure S1.** Detailed view of the homology model showing H-bonds (dotted lines, numbers indicate length in Angstroms) between side chain of Glu291 (helix H, magenta) and main chains of Val540 and Ser541 (helix P, transparent pink).

**References:**

1. Brugnoni R, Kapetis D, Imbrici P, Pessia M, Canioni E, et al. (2013) A large cohort of myotonia congenita probands: novel mutations and a high-frequency mutation region in exons 4 and 5 of the CLCN1 gene. J Hum Genet 58: 581-587.

2. Mazon MJ, Barros F, De la Pena P, Quesada JF, Escudero A, et al. (2012) Screening for mutations in Spanish families with myotonia. Functional analysis of novel mutations in CLCN1 gene. Neuromuscul Disord 22: 231-243.

3. Fialho D, Schorge S, Pucovska U, Davies NP, Labrum R, et al. (2007) Chloride channel myotonia: exon 8 hot-spot for dominant-negative interactions. Brain 130: 3265-3274.

4. Ivanova EA, Dadali EL, Fedotov VP, Kurbatov SA, Rudenskaya GE, et al. (2012) The spectrum of CLCN1 gene mutations in patients with nondystrophic Thomsen's and Becker's myotonias. Russ J Genet+ 48: 952-961.

5. Sun C, Tranebjaerg L, Torbergsen T, Holmgren G, Van Ghelue M (2001) Spectrum of CLCN1 mutations in patients with myotonia congenita in Northern Scandinavia. Eur J Hum Genet 9: 903-909.

6. Colding-Jorgensen E, Dun OM, Schwartz M, Vissing J (2003) Decrement of compound muscle action potential is related to mutation type in myotonia congenita. Muscle Nerve 27: 449-455.

7. Trip J, Drost G, Verbove DJ, van der Kooi AJ, Kuks JBM, et al. (2008) In tandem analysis of CLCN1 and SCN4A greatly enhances mutation detection in families with non-dystrophic myotonia. Eur J Hum Genet 16: 921-929.
